# An Assessment of Antibacterial Activity of Four Endodontic Sealers on *Enterococcus faecalis* by a Direct Contact Test: An In Vitro Study

**DOI:** 10.5402/2012/989781

**Published:** 2012-07-19

**Authors:** Lavanya Anumula, Swaroop Kumar, Venkata Suneel Kumar, Chandra Sekhar, Murali Krishna, Rama Mohan Pathapati, Prathi Venkata Sarath, Yamini Vadaganadam, Rakesh Kumar Manne, Srinath Mudlapudi

**Affiliations:** ^1^Department of Conservative Dentistry and Endodontics, RIMS Government Dental College, Andhra Pradesh, Kadapa 516004, India; ^2^Department of Conservative Dentistry and Endodontics, Narayana Dental College and Hospital, Andhra Pradesh, Nellore 524003, India; ^3^Department of Oral Medicine & Radiology, Narayana Dental College and Hospital, Andhra Pradesh, Nellore 524002, India; ^4^Department of Conservative Dentistry & Endodontics, KLR's Lenora Institute of Dental Sciences, Andhra Pradesh, Rajahmundry 533101, India; ^5^Department of Pharmacology, Narayana Medical College and Hospital, Andhra Pradesh, Nellore 524002, India; ^6^Department of Pedodontics, Narayana Dental College and Hospital, Andhra Pradesh, Nellore 524002, India; ^7^Department of Orthodontics, Narayana Dental College and Hospital, Andhra Pradesh, Nellore 524002, India

## Abstract

*Aim*. To evaluate the antibacterial activity of four endodontic sealers on *Enterococcus faecalis* by a direct contact test. *Material and Methods*. *Enterococcus faecalis* was used as a test organism. Direct contact test which is based on measuring the effect of close contact between test bacteria and tested material on the kinetics of bacterial growth was performed to overcome the disadvantages of agar diffusion test. The sealers tested were zinc oxide eugenol-based sealer, glass-ionomer-based sealer, polydimethyl-siloxane-based sealer, and urethane dimethacrylate resin-based sealer. Data was collected by recording the optical density with the help of a spectrophotometer. *Results*. The sealers exhibited different inhibitory effects. The results obtained were subjected to statistical analysis by Kruskal Wallis analysis of variance and Dunn's multiple comparison test. Group comparison showed very highly significant difference between the groups. *Conclusion*. Zinc oxide eugenol-based sealer was the most effective and urethane dimethacrylate resin-based sealer was the least effective against *Enterococcus faecalis*, whereas glass-ionomer-based and polydimethyl-siloxane-based sealers were effective only for a short period. Inhibition of the bacterial growth is related to the direct contact of the microorganism with the sealer.

## 1. Introduction

Bacteria or their byproducts are considered to be the primary etiological agents of pulpal necrosis and periapical lesions [1]. The main objective of endodontic therapy is therefore to eliminate bacteria from the infected root canal [2].

The majority of the bacteria found in the root canal system may be eliminated by the biomechanical cleaning and shaping of the root canal space. Failure of the root canal treatment is the result of microorganisms persisting in the apical portion of the root canal system, even in well-treated teeth [3] due to the anatomical complexities of many root canals, such as dentinal tubules, ramifications, deltas, and fins which cannot be sufficiently cleaned, even after meticulous mechanical procedures.

*Enterococcus faecalis* is a recalcitrant candidate among the many causative agents of failed endodontic treatment [4]. 38% of the failed root canal systems were contaminated with *Enterococcus faecalis* [5]. Chronic failure of an endodontically treated tooth is due to ability of *E. faecalis* to bind to the collagen of the dentinal tubule and remain viable within the tubules [6]. These microorganisms have the ability to grow even in a low-nutrient environment and can survive in the root canals as a monoinfection [7]. Eradication of *E. faecalis* from the root canal with chemomechanical preparation using disinfecting irrigants and antibacterial dressings is difficult.

Most currently used root canal obturating materials do not possess a long-lasting perfect seal with the root canal wall. Microleakage remains a clinical problem and a possible cause of failure of endodontic therapy [8]. The use of sealers with antibacterial properties may be advantageous especially in clinical situations of persistent or recurrent infection [9]. The endodontic sealers have been shown to offer the greatest antimicrobial effects immediately after spatulation, following which there will be a gradual loss of antimicrobial effects over time [10]. The antibacterial property of the newly introduced resin-based sealers, polydimethyl-siloxane-based (Gutta Flow), and urethane dimethacrylate resin-based sealer, (Endo Rez) is questioned.

The agar diffusion test is the most commonly used technique to assess antibacterial activity of sealers. But it has many limitations as it is dependent on diffusion and physical properties of tested materials. Direct contact test was developed by Weiss et al. [10]. The antibacterial activity of the endodontic sealers can be evaluated by measuring the kinetics of bacterial growth [11]. Even insoluble materials can be tested with this quantitative assay. To this purpose, we evaluated the in vitro antimicrobial activity of four endodontic sealers (zinc oxide eugenol sealer, Ketac Endo Applicap, Gutta Flow, and Endo Rez) on *Enterococcus faecalis* by direct contact test.

## 2. Material and Methods

In our study, we used the *Enterococcus faecalis* (ATCC 35550) strain which was grown aerobically on frozen stock cultures of brain heart infusion (BHI) broth at 37°C. Cells were harvested by centrifugation and resuspended in fresh medium. Inoculum was prepared by the resuspension of washed cells to predetermined optical densities which relate to known concentrations.

The tested materials were categorized as follows.

 Group I: zinc oxide eugenol sealer (DPI).
 Group II: glass ionomer sealer (Ketac Endo Applicap).
 Group III: polydimethyl siloxane based (Gutta Flow).
 Group IV: resin based (Endo Rez).
 Group V: control-bacterial suspension in the absence of sealer.

The sealers were prepared in strict compliance with the manufacturers' recommendation.

### 2.1. Direct Contact Test (DCT) (Figure 1)

The direct contact test, a turbidometric determination of bacterial growth kinetics, was monitored in each well every 30 min for 16 hours using a spectrophotometer (Stat fax 2100 reader M/s Awareness Technology, Inc., USA) at 600 nm at 37°C. 96 wells of a microtitre plate were used out of which 8 wells were utilized per sealer of which 4 were designated as “A” wells (with the sealer) and the other 4 as “B” wells (without the sealer). The “A” wells were held vertically, that is, the plate's surface was maintained perpendicular to the floor plane and the side wall was coated with freshly mixed tested material. Even and thin coating was achieved by using a small size round ended dental instrument, such as a cavity liner applicator. Special care was taken to avoid the material's flow to the bottom of the well, which would interfere with the path of light through the microplate well and result in false readings. After 20 min, a 10 *μ*L bacterial suspension containing 10^6^ bacteria was placed on the test material. The plate was held in a vertical position, and wells were inspected for evaporation of the suspension's liquid, which occurred within 1 hr at 37°C. This ensured direct contact between bacteria and tested material. Brain heart infusion broth (245 *μ*L) was added to each of these A wells and gently mixed for 2 min. 15 *μ*L of broth was then transferred from A wells to an adjacent set of B wells containing fresh medium (215 *μ*L). This resulted in two sets of 4 wells for each tested material containing an equal volume of liquid medium so that bacterial out growth could be monitored both in the presence and in the absence of the tested material. Following the outgrowth of the microorganism in the presence of the tested material (Group A wells) is equivalent to measuring both the direct contact effect and the effect of those components which are capable of diffusing into the liquid medium, whereas following bacterial growth in the absence of the tested materials (Group B wells) measures the effect of the direct contact incubation period only. 4 uncoated wells in the same microtiter plate served as positive control, that is, identical bacterial inoculums were placed on the side wall of the uncoated wells and processed as the experimental A and B wells. The whole experiment was carried out under aseptic conditions and was repeated six times to ensure reproducibility.

## 3. Statistical Analysis

Data were recorded then plotted and statistically analyzed using Kruskal Wallis test followed by Dunn's post hoc analysis.

## 4. Results

**Figure 2:**
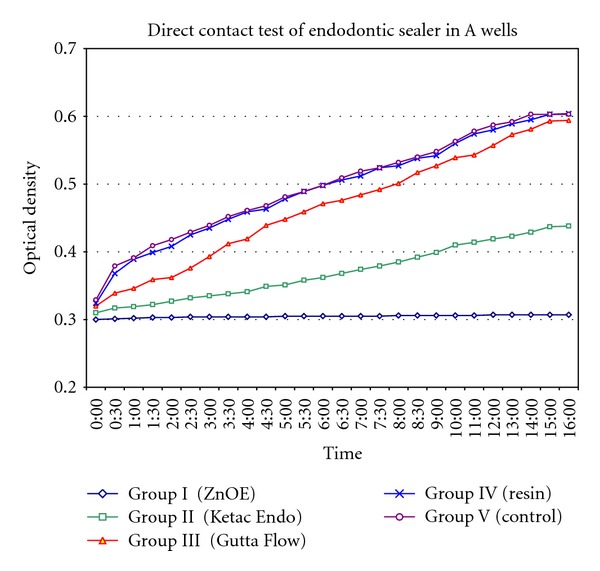
Direct contact test of endodontic sealer in “A” wells.

**Figure 3:**
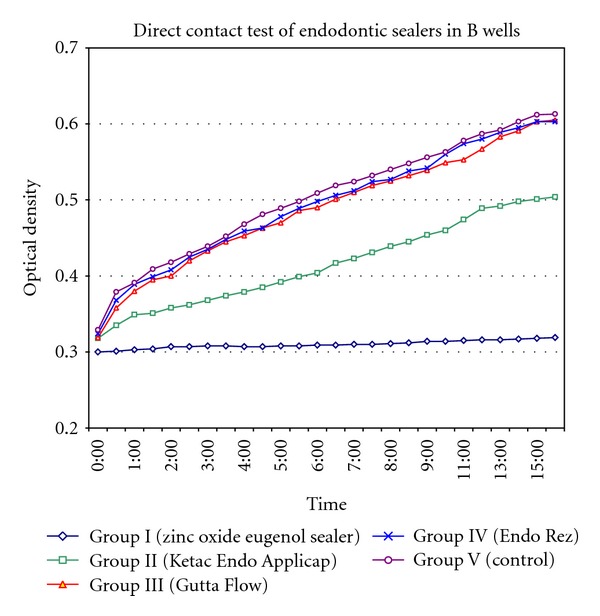
Direct contact test of endodontic sealers in “B” wells.

The results of the direct contact test of endodontic sealers for the time period of 16 hours are shown in Figures 2 and 3. Each point on the growth curve is the average of optical density measurements in 4 wells at any given time (0–16 hrs). In both wells, Group I showed constant and complete inhibition of the bacterial growth throughout the incubation period, Group II showed inhibition of the bacteria in the first 10 hours and slowly decreased in efficiency, Group III inhibited bacteria only in the first 3 hours followed by a brisk decrease only in A wells where as in B wells there was no inhibition of bacterial growth, Group IV did not show any antibacterial activity, and Group V showed continuous growth of microorganism. The mean and standard deviations of OD of all 0–16 time points were shown in Table 1.

The intergroup comparisons between groups for both A and B wells were shown in Table 2. It can be noticed that on comparison to control group in both A and B wells, Group I and Group II showed significant difference in overall bacterial kinetics. However, such a difference was not observed with Groups III and IV.

## 5. Discussion

The golden rule in the practice of endodontology is to debride and obturate the canals as efficiently and three dimensionally as possible and to prevent subsequent reinfection. However, part of the root canal space often remains untouched during chemomechanical preparation regardless of the technique and instruments employed [12, 13]. Obturating the root canal system using a sealer with antibacterial properties may be advantageous especially in clinical situations of persistent or recurrent infections [9].

 These antibacterial effects of sealers may explain the minute difference in the success rate of root canal treatment completed in one or more appointments [14, 15]. Most important requirements of sealers are biocompatibility, excellent seal, adequate adhesion, and antimicrobial property. Rappaport, 1964, stressed on the fact that “the ideal root canal cement should be bactericidal” [16].

In this study, direct contact test (DCT) has been used to assess the antibacterial activity which has many advantages over agar diffusion test [10, 11, 17]. The present study utilizes and proves direct contact test as an appropriate method of testing antimicrobial activity as in accordance with other studies [11, 17–19].

Zinc oxide eugenol has a long time record and is utilized as a standard sealer which has shown the maximum antibacterial activity [20–22]. In this study the antibacterial activity glass-ionomer-based, urethane dimethacrylate resin-based, and polydimethyl-siloxane-based sealers were evaluated and compared.

In our study, also zinc oxide eugenol sealer showed a complete inhibition of the bacterial growth throughout the incubation period.

Ketac Endo Applicap (glass-ionomer-based sealer) demonstrated a lower antibacterial activity when compared to that of zinc oxide eugenol-based sealers. It showed antibacterial activity only for a short time (10 hours). GIC has strong antimicrobial property, the mechanism of which is probably a function of both fluoride release and low pH [23], although additional factors like release of zinc ions and better homogenous structure may be involved [24, 25]. The release of fluoride from the glass ionomer materials is pH dependent, “burst effect” of fluoride for the first and second day followed by a significant decrease. This may explain the initial antibacterial effect of GIC-based sealer. Fluoride can have three effects on bacteria, that is, inhibition of metabolism, inhibition of growth, and bacterial death. Growth of inhibition was directly related to the amount of fluoride ions released. A direct bactericidal effect does not occur from fluoride released, since the amount of fluoride released is too low.

Gutta Flow (polydimethyl siloxane) based endodontic sealer showed a slight antibacterial activity for the first 3 hours which drastically reduced with time, whereas, in “B” wells, there was no inhibition of bacterial growth. The antibacterial activity is attributed to the nanosilver present in the sealer which is used as a preservative. This may be related to the oligodynamic effect, that is, high affinity of metal ions (silver) to cellular proteins that combine with sulfur groups and denature the proteins [26].

Endo Rez (urethane dimethacrylate resin) based endodontic sealer did not show any antibacterial activity against *Enterococcus faecalis*, which may be due to the absence of an antibacterial component in its composition.

## 6. Conclusion

The sealers evaluated in this study showed different inhibitory effects during the time interval studied. Zinc oxide eugenol-based sealer was the most effective and urethane dimethacrylate resin-based sealer was the least effective against *Enterococcus faecalis*. The antibacterial property of the endodontic sealers gradually decreased over time. Inhibition of the bacterial growth is related to the direct contact of the microorganism with the sealer. Hence, the incorporation of antimicrobial components into root canal sealers may become an essential factor in preventing the regrowth of residual bacteria and control of bacterial reentry into the root canal space.

## Figures and Tables

**Figure 1:**
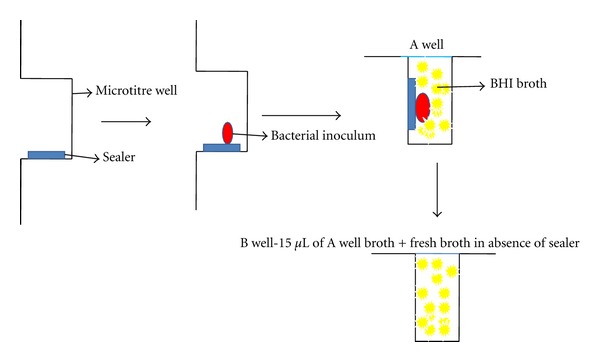
Schematic representation of DCT.

**Table 1:** Showing bacterial kinetics (mean optical density).

Groups	Well A	Well B
Group I (ZnOE)	0.30 ± 0.01	0.31 ± 0.01
Group II (Ketac Endo)	0.37 ± 0.04	0.41 ± 0.06
Group III (Gutta Flow)	0.46 ± 0.08	0.49 ± 0.08
Group IV (resin)	0.49 ± 0.07	0.49 ± 0.08
Group V (control)	0.49 ± 0.07	0.50 ± 0.09

**Table 2:** Showing intergroup statistical significance of bacterial growth kinetics.

Dunn's multiple comparison test	A wells	B wells
Group I (ZnOE) versus Group II (Ketac Endo)	*P* < 0.01	*P* < 0.001
Group I (ZnOE) versus Group III (Gutta Flow)	*P* < 0.001	*P* < 0.001
Group I (ZnOE) versus Group IV (resin)	*P* < 0.001	*P* < 0.001
Group I (ZnOE) versus Group V (control)	*P* < 0.001	*P* < 0.001
Group II (Ketac Endo) versus Group III (Gutta Flow)	*P* < 0.05	*P* > 0.05
Group II (Ketac Endo) versus Group IV (resin)	*P* < 0.001	*P* < 0.05
Group II (Ketac Endo) versus Group V (control)	*P* < 0.001	*P* < 0.05
Group III (Gutta Flow) versus Group IV (resin)	*P* > 0.05	*P* > 0.05
Group III (Gutta Flow) versus Group V (control)	*P* > 0.05	*P* > 0.05
Group IV (resin) versus Group V (control)	*P* > 0.05	*P* > 0.05
